# Endothelial Mechanistic Target of Rapamycin Activation with Different Strains of *R. rickettsii*: Possible Role in Rickettsial Pathogenesis

**DOI:** 10.3390/microorganisms12020296

**Published:** 2024-01-30

**Authors:** Abha Sahni, Jessica Alsing, Hema P. Narra, Michelle Montini, Yasim Zafar, Sanjeev K. Sahni

**Affiliations:** Department of Pathology, University of Texas Medical Branch, Galveston, TX 77555-0609, USA; jealsing@utmb.edu (J.A.); hpnarra@utmb.edu (H.P.N.); mamontin@utmb.edu (M.M.); yazafar@utmb.edu (Y.Z.)

**Keywords:** endothelial cells, mTOR, *Rickettsia*, autophagy, inflammation

## Abstract

*Rickettsia rickettsii* is an obligate intracellular pathogen that primarily targets endothelial cells (ECs), leading to vascular inflammation and dysfunction. Mechanistic target of rapamycin (mTOR) regulates several cellular processes that directly affect host immune responses to bacterial pathogens. Here, we infected ECs with two *R. rickettsii* strains, avirulent (Iowa) and highly virulent Sheila Smith (SS) to identify differences in the kinetics and/or intensity of mTOR activation to establish a correlation between mTOR response and bacterial virulence. Endothelial mTOR activation with the highly virulent SS strain was significantly higher than with the avirulent Iowa strain. Similarly, there was increased LC3-II lipidation with the virulent SS strain compared with the avirulent Iowa strain of *R. rickettsii*. mTOR inhibitors rapamycin and Torin2 significantly increased bacterial growth and replication in the ECs, as evidenced by a more than six-fold increase in rickettsia copy numbers at 48 h post-infection. Further, the knockdown of mTOR with Raptor and Rictor siRNA resulted in a higher rickettsial copy number and the altered expression of the pro-inflammatory cytokines interleukin (IL)-1α, IL-6, and IL-8. These results are the first to reveal that endothelial mTOR activation and the early induction of autophagy might be governed by bacterial virulence and have established the mTOR pathway as an important regulator of endothelial inflammation, host immunity, and microbial replication.

## 1. Introduction

Rocky Mountain spotted fever (RMSF) is an infectious disease caused by the Gram-negative, obligate intracellular bacterium *Rickettsia rickettsii* (*R. rickettsii*). Rickettsiae are transmitted to humans by the bites of infected ticks, lice, fleas, and mosquitoes [1,2]. After a bite, the bacteria enter the bloodstream and target the microvascular endothelial cells (ECs), where they establish themselves and multiply, leading to inflammation and edema, collectively termed vasculitis. *R. rickettsii* infection primarily affects blood vessels and tissues of the lungs, brain, and spinal cord (central nervous system) and can affect other organs like the heart, kidneys, and spleen [1,3]. In the absence of proper and timely treatment, individuals usually develop potentially life-threatening complications because of tissue and organ injuries and dysfunction [4]. Rickettsial infections are widespread throughout all continents except Antarctica, but the severity of the disease varies significantly based on the virulence of the pathogen. Therefore, a detailed analysis of host–pathogen interactions is critically important for advancing our knowledge of disease pathogenesis and the advancement of treatment options.

The master regulator, mammalian target of rapamycin (mTOR), comprises two distinct intracellular protein complexes, mTORC1 and mTORC2, which exhibit distinct functions. mTORC1 is rapamycin-sensitive and primarily involved in regulating protein synthesis, lipid metabolism, and organelle biogenesis, while mTORC2 is rapamycin-insensitive and involved in the regulation of the actin cytoskeleton, metabolism, and cell survival [5]. mTORC1 is composed of several proteins with a catalytic subunit and a unique protein Raptor (regulatory-associated protein of mTOR), representing the core components [6]. In response to stimulus, mTORC1 modulates the activity of various signaling pathways, including ribosomal S6 kinase (S6K) and its downstream target, ribosomal S6 protein. mTORC2 has Rictor as a core component and has been shown to affect the cellular cytoskeleton and Akt phosphorylation [7,8]. During infection, intracellular bacteria solely depend on nutrition to survive and hijack host machinery controlling the cellular metabolic processes [9]. In addition, these pathogenic bacteria can subvert host metabolism by targeting mTOR complexes for their replication and the establishment of infection [10,11]. In contrast, host cells utilize mTOR to facilitate pathogen clearance by regulating innate and adaptive immune responses [12]. The drugs that strategically target mTOR-mediated pathogen replication may alter the outcome of host–pathogen interactions. The virulence factors of the pathogens utilize mTOR to manipulate the host metabolic machinery to modify immunological outcomes for their own survival.

The mTOR pathway regulates several essential cellular functions like metabolism and autophagy. Autophagy is a conserved eukaryotic cellular recycling and trafficking process where cytosolic material is engulfed within autophagosomes and degraded by lysosomes [13]. Upon infection, autophagy is induced by several host factors and signaling pathways, leading to pathogen encapsulation within autophagosomes that ultimately fuse with lysosomes, causing the degradation and, ultimately, clearance of the pathogen [13]. Conversely, pathogens can hijack host autophagic machinery to increase their intracellular replication and further invasion potential. We recently demonstrated that both mTORC1 and mTORC2 are activated in *R. rickettsii*-infected endothelial cells [14]. Here, we further investigated if the activation of mTOR is dependent on virulence, and we have utilized the avirulent (Iowa) and highly virulent (SS) strains of *R. rickettsii* to identify differences in the intensity/kinetics of mTOR to establish a correlation between the host cell’s mTOR response and rickettsial virulence. We demonstrated that mTOR activation is an important host defense mechanism that interferes with the replication of intracellular rickettsiae by inhibiting the process of autophagy and regulating inflammation.

## 2. Materials and Methods

### 2.1. Cell Culture and Infection

Human dermal microvascular endothelial cells (HMECs) represented by an immortalized cell line, HMEC-1, were obtained from the Centers for Disease Control and Prevention (Atlanta, GA, USA) and grown in MCDB-131 medium (Caisson Labs, Smithfield, UT, USA, catalog #MBL02) containing FBS (10% *v*/*v*), epidermal growth factor (10 ng/mL, ThermoFisher, Waltham, MA, USA, catalog #PHG0311), hydrocortisone (1 μg/mL, Sigma, Livonia, MI, USA, catalog #H0888), and L-glutamine (10 mM, ThermoFisher, catalog #A2916801), as described previously [15]. *R. rickettsii* Iowa strain was obtained from Dr. Ted Hackstadt, NIH/NIAID, Rocky Mountain Laboratories, Hamilton, MT. Both the Sheila Smith (SS) and Iowa strains of *R. rickettsii* were grown in Vero cells prepared via gentle cell lysis using glass beads and purified via centrifugation, and aliquoted stocks of ~0.5 mL were kept frozen at −80 °C to avoid repeated freeze–thaw cycles, which may result in the loss of rickettsial viability. Infectivity titers of purified stocks were measured with two separate methods, one via citrate synthase (gltA)-based quantitative PCR and the second with a plaque assay using our established lab protocols [16,17].

The doses of the *R. rickettsii* strains were normalized to have a similar number of bacteria at the time of infecting the cells. We measured the rickettsia copy number at 3 and 24 h post-infection with both the Iowa and SS strains of *R. rickettsii* and adjusted the dose of the bacteria to have similar numbers. The normalized dose at the time of infection excludes the possibility of a difference in the mTOR activation given the difference in the number of bacteria used at the time of infection.

Confluent endothelial cell monolayers (80–90% confluence) were either mock-infected or infected with both strains of *R. rickettsii* according to published protocols [18]. Briefly, *R. rickettsii* stocks (normally 1 × 10^7^ to 5 × 10^7^) were diluted in culture medium, and endothelial cells were infected with approximately 6 × 10^4^ Pfu per cm^2^ of cell culture area. Cells were rocked at room temperature for 15 min and then incubated at 37 °C with 5% CO_2_. At 3 and 24 h post-infection, total cell lysates were prepared by washing the cells with phosphate-buffered saline (PBS), gently scraping in a lysis buffer containing protease and phosphatase inhibitor cocktail (ThermoFisher, catalog #78442).

### 2.2. mTOR Inhibition

Rapamycin (catalog #S1039) and Torin2 (catalog #S2817) were purchased from Selleckchem and resuspended in the minimum amount of DMSO to dissolve the compounds and prepare the initial 20 mM stocks. Confluent endothelial cells were treated with rapamycin (5 nM) and Torin2 (12.5 nM) for 2 h to inhibit mTOR activity. The medium was then removed, and the cells were infected with *R. rickettsii* for 48 h. Endothelial cells treated with DMSO were used as a mock control. In some experiments, mTORC1-specific Raptor siRNA (25 or 50 nM, catalog #sc-44069), mTORC2-specific Rictor siRNA (25 or 50 nM, catalog #sc-61478), or control siRNA (50 nM, catalog #sc-44230) (Santa Cruz Biotechnology, Dallas, TX, USA) were transfected into endothelial cells using Lipofectamine RNAiMAX (ThermoFisher, catalog #13778075) according to the manufacturer’s instructions for 48 h prior to infection with *R. rickettsii*, and total cell lysates were prepared as described above.

### 2.3. Western Blotting

Total protein lysate samples were analyzed on 8, 10, or 15% polyacrylamide gel based on the protein size and transferred onto a PVDF membrane (BioRad, Hercules, CA, USA), followed by probing the blots with primary antibodies and then horseradish peroxidase (HRP)-linked secondary antibodies for chemiluminescence-based detection. We used phospho-p70 S6 kinase (Thr412/Ser424, catalog #9204), phospho-Akt (Ser473, catalog #4058), phospho-mTOR (Ser2448, catalog #2971), total p70 S6 kinase (catalog #9202), total Akt (catalog #4691), total mTOR (catalog #2983), LC3II (catalog #2775), Raptor (catalog #2280), and Rictor (catalog #9476) protein expression antibodies from Cell Signaling Technology. The blots were stripped and reprobed with an α-tubulin antibody to account for variations in the protein loading.

### 2.4. RNA Isolation and qPCR

Total RNA was isolated from mock- or rickettsia-infected ECs using TRI^®^ Reagent (Molecular Research Center, Cincinnati, OH, USA, catalog #TR118) followed by the preparation of cDNA using a cDNA synthesis kit (ThermoFisher, catalog #4368814). PCR reactions were performed with a StepOnePlus^TM^ thermal cycler (Applied Biosystems, Foster City, CA, USA) using cDNA and specific primers for each cytokine: IL-1α, IL-6, and IL-8 (Table S1). Cytokine mRNA expression was normalized to GAPDH, and relative expression was calculated using the ^ΔΔ^CT method, as described previously [18]. To determine the rickettsia copy number, total DNA was isolated using the DNeasy Blood and Tissue Kit (Qiagen, Germantown, MD, USA, catalog #69504) according to the manufacturer’s instructions and quantified with a spectrophotometer (ThermoFisher). Quantitative PCR was performed using the primer pair RR190.547F and RR190.701R for rickettsial outer membrane protein A (rOmpA) for spotted-fever-group rickettsiae [19].

### 2.5. Densitometry and Statistical Analysis

The densitometric analysis was performed as described previously [14]. Briefly, the band intensity was calculated using the Image Studio Lite software and normalized to α-tubulin. Expression levels in mock-infected controls were assigned a value of 1 for comparison with infected or treated experimental conditions. Each experiment was performed at least three times with replicates, and the results are presented as the mean ± standard error of the mean (SEM). Statistical significance between the control and infected/treated groups was evaluated with Student’s *t*-test and/or one-way analysis of variance (ANOVA) with Dunnett’s post-test using GraphPad Prism 9.5. The significant changes threshold was set at a *p*-value of ≤0.05.

## 3. Result

**Figure 1:**
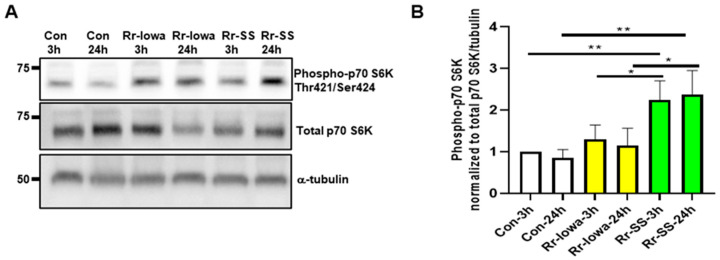
Endothelial mTORC1 activation during infection with *R. rickettsii* virulent strain (Sheila Smith (SS)) and avirulent strain (Iowa). (**A**) Confluent ECs were infected with the avirulent (Iowa) or virulent (SS) strains of *R. rickettsii* at 3 and 24 h. Control cells were mock-infected at the same time points. Total protein lysates were then prepared and subjected to Western blot analysis using antibodies against phospho- and total p70 S6 kinase to determine the activation of mTORC1. The α-tubulin antibody was used as a loading control. The results of a representative blot (*n* ≥ 5) are shown. (**B**) A densitometric analysis of phospho-p70S6 kinase during rickettsial infection is shown, where the band intensities of phospho-p70S6K are normalized to total p70S6K and α-tubulin. The data represent the mean ± standard error of the mean (SEM) of at least three independent experiments. (* = *p* ≤ 0.05, ** = *p* ≤ 0.01).

mTOR is a central regulator of metabolism, acts as a nutrient sensor for intracellular pathogens, and regulates innate and adaptive immune responses [8,20,21]. To determine the endothelial mTORC1 activation during infection with the highly virulent SS and avirulent Iowa strains of *Rickettsia rickettsii*, Western blot analysis was performed using the known substrate p70S6 kinase (p70S6K). The bacterial dose of both strains was normalized to have a similar number of bacteria at the time of infection. Significantly higher levels of phospho-p70S6K were observed in response to the highly virulent SS strain compared with the avirulent Iowa strain of *R. rickettsii* at both 3 and 24 h post-infection (Figure 1A). The quantitative densitometric analysis presented in Figure 1B clearly demonstrates an about two-fold increase in phospho-p70S6K (Threonine 412/Serine 424) expression in the SS strain compared with the Iowa strain of *R. rickettsii*-infected cells at both 3 and 24 h post-infection (Figure 1B). The data indicate the differential activation of endothelial mTORC1 in response to the avirulent Iowa and highly virulent SS strains of *R. rickettsii* infection.

**Figure 2:**
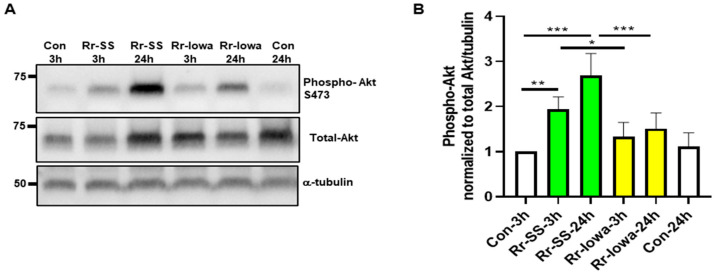
Endothelial mTORC2 activation during infection with *R. rickettsii* virulent strain (SS) and avirulent strain (Iowa). (**A**) Confluent ECs were infected with Iowa and SS strains of *R. rickettsii* at 3 and 24 h. Control cells were mock-infected at the same time points. Total protein lysates were prepared and subjected to Western blotting using antibodies against phospho- and total AKT. The α-tubulin antibody was used as a loading control. The results of a representative blot (*n* = 5) are presented. (**B**) A quantitation of the phospho-Akt bands normalized to total Akt and tubulin during EC infection with both strains of *R. rickettsii* is shown. The data represent the mean ± SEM of three different experiments. (* = *p* ≤ 0.05, ** = *p* ≤ 0.01, *** = *p* ≤ 0.001).

To determine the mTORC2 activation in response to both strains of *R. rickettsii*-infected ECs, we measured the phosphorylation status of the mTORC2 substrate Akt on Serine 473 at 3 and 24 h post-infection. The results reveal a significantly higher expression of phospho-Akt with the highly virulent SS strain compared with the avirulent Iowa-strain-infected cells (Figure 2A). Endothelial phospho-Akt levels in response to the highly virulent SS strain displayed an about two-fold or higher increase in infected cells at 3 h and 24 h post-infection compared with the mock-infected endothelial cells (Figure 2A). In contrast, no significant increase was observed in the phospho-Akt levels in endothelial cells infected with the avirulent Iowa strain of *R. rickettsii*. A densitometric analysis of phospho-Akt quantitation normalized to total and α-tubulin with both strains of *R. rickettsii*-infected cells is shown in Figure 2B. Together, these results demonstrate the significantly higher expression of mTORC2 with the highly virulent SS strain compared with the Iowa strain of *R. rickettsii*-infected endothelial cells.

**Figure 3:**
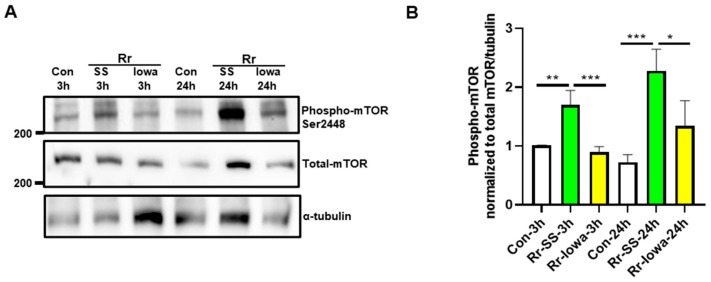
Endothelial mTOR phosphorylation during infection with virulent strain (SS) and avirulent strain (Iowa) *R. rickettsii*. (**A**) ECs were infected with *R. rickettsii* (SS and Iowa strain) at 3 and 24 h. Control cells were mock-infected at the same time points. Cell lysates were then prepared and subjected to Western blotting using phospho- and total mTOR antibodies. For loading control, the α-tubulin antibody was used. The results of a representative blot (*n* ≥ 3) are shown. (**B**) A densitometric analysis of the phospho-mTOR normalized to total mTOR and tubulin in *R. rickettsii* (SS and Iowa)-infected ECs is shown. The results represent the mean ± SEM of three separate experiments. (* = *p* ≤ 0.05, ** = *p* ≤ 0.01, *** = *p* ≤ 0.001).

Next, we measured the direct phosphorylation of mTOR itself on Serine 2448 with both strains of *R. rickettsii*. As expected, we noticed a significantly higher increase with the highly virulent SS strain at both 3 h (1.7 ± 0.3-fold) and 24 h (2.3 ± 0.5-fold) post-infection in mTOR phosphorylation compared with the mock-infected controls (Figure 3A). There was no significant increase in phospho-mTOR observed in the endothelial cells infected with the avirulent Iowa strain of *R. rickettsii* at either time point. A densitometric analysis of phospho-mTOR in mock- and *R. rickettsii* (SS and Iowa)-infected endothelial cells from three separate experiments is depicted in Figure 3B. These results demonstrate that the infection of host ECs with the highly pathogenic SS strain induces more mTOR activation compared with the avirulent Iowa strain of *R. rickettsii*.

**Figure 4:**
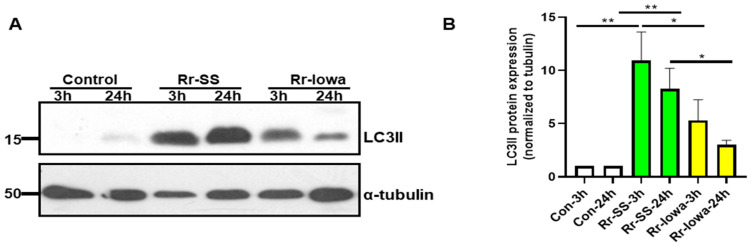
LC3II lipidation in ECs infected with avirulent (Iowa) and highly virulent (SS) strain of *R. rickettsii.* (**A**) ECs were infected with the SS or Iowa strain of *R. rickettsii* at 3 and 24 h. Control cells were mock-infected at the same time points. Total cell lysates were prepared after each time point and subjected to Western blotting using the LC3II antibody. The blots were probed with the α-tubulin antibody to normalize the loading variations. (**B**) A densitometric analysis of the bands of LC3II lipidation during infection with SS and Iowa strains of *R. rickettsii* is shown. (* = *p* ≤ 0.01, ** = *p* ≤ 0.001).

Various published reports demonstrate the important role of mTOR in the regulation of the autophagic process [5,22]. Therefore, we next investigated the initial host endothelial cells’ autophagic responses to both strains of *R. rickettsii* upon infection and measured the initial step of autophagy by analyzing the lipidation of the LC3II protein. Cells infected with the highly virulent SS strain of *R. rickettsii* displayed significantly higher LC3II lipidation both at 3 and 24 h post-infection compared with mock-infected controls. In contrast, only a modest increase was observed with the avirulent Iowa strain at 3 h but not at 24 h post-infection (Figure 4A). Again, a quantitation of the densitometric analysis of LC3II lipidation in mock- and *R. rickettsii* (SS and Iowa)-infected ECs from three separate experiments is depicted in Figure 4B. These results clearly demonstrate that the highly virulent strain SS initiates the autophagic response in endothelial cells by activating mTOR in contrast to the avirulent strain of *R. rickettsii*.

**Figure 5:**
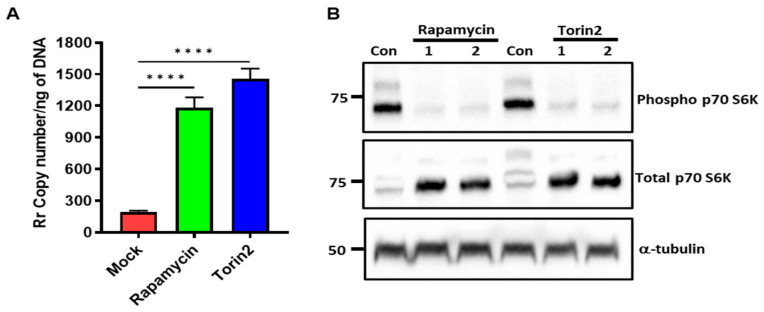
Effect of rapamycin and Torin2 on rickettsial replication. ECs were treated with rapamycin (5 nM) or Torin2 (12.5 nM) along with a mock treatment (DMSO) for 2 h. The medium was then removed, and cells were infected with *R. rickettsii* (SS) at 48 h. DNA was isolated, and rickettsial copy number was measured (**A**) Total protein lysates were prepared and subjected to Western blotting using antibodies against phospho- and total p70S6 kinase. α-tubulin antibody was used as a loading control. (**B**) The results of a representative blot (*n* ≥ 3) are shown. 1 and 2 represent two separate samples treated with either rapamycin or Torin2. Statistically significant changes are shown (**** = *p* ≤ 0.0001).

Intracellular bacterial pathogens utilize multiple strategies, like evading the cellular autophagic process or hijacking host cell functions for their survival and replication [23]. Both the Iowa and SS strains of *R. rickettsii* exhibit identical replication patterns, similar abilities to form actin tails, and indistinguishable plaque sizes, suggesting that the rates of growth and cell-to-cell spread are similar between the two strains [24]. We also determined the intracellular growth pattern of both SS and Iowa in ECs at 24 and 48 h and observed that their growth rates are nearly identical (Supplementary Figure S1); therefore, we only used SS for our further experiments. To investigate the involvement of mTOR in rickettsial replication, ECs were treated with 5 nM of rapamycin or 12.5 nM of Torin2 prior to infecting the cells with *R. rickettsii* (SS). After 48 h, DNA was prepared to determine the rickettsia copy numbers in the mTOR-inhibited cells. The results clearly demonstrate a 6.2 ± 0.6-fold increase in the rickettsial copy number with the rapamycin treatment and a 7.7 ± 0.7-fold increase with the Torin2 treatment in direct comparison with the mock-treated ECs (Figure 5A). Next, we determined if rapamycin and Torin2 inhibit mTORC1 by measuring the phosphorylation of p70S6K, and the results indicate that there is a clear inhibition of p70S6 phosphorylation in both rapamycin- and Torin2-treated cells compared with mock-treated endothelial cells (Figure 5B). These results indicate that pathogenic rickettsiae utilize mTOR for their replication inside host cells.

**Figure 6:**
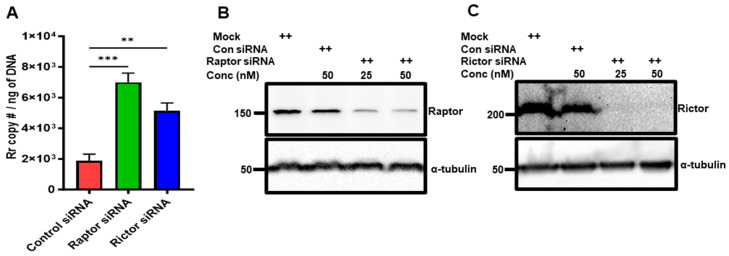
Effect of Raptor and Rictor siRNA on rickettsial replication. ECs were transfected with Raptor or Rictor siRNA (25 or 50 nM), along with control siRNA (50 nM), using Lipofectamine RNAiMAX 24 h prior to infection with *R. rickettsii* (SS) for 48 h. DNA was isolated, and rickettsial copy number was measured (**A**). Total protein lysates were prepared and subjected to Westen blot using antibodies against Raptor and Rictor to determine the extent of Raptor knockdown (**B**) and Rictor knockdown (**C**). The α-tubulin antibody was used as a loading control, and a representative blot (*n* ≥ 3) is shown. In some experiments, (**C**) statistically significant changes are shown as ** = *p* ≤ 0.001 and *** = *p* ≤ 0.0001.

Next, we used the RNAi approach for the selective inhibition of mTORC1 and mTORC2 to determine if one or both complexes regulate rickettsial replication. ECs were transfected with Raptor or Rictor siRNA along with control siRNA 48 h prior to infection with *R. rickettsii* (SS). DNA was prepared after 48 h post-infection and the rickettsia copy number was measured. The results indicate that inhibiting both mTOR complexes increased rickettsial replication, but the effect was more pronounced in inhibiting mTORC1 using Raptor siRNA compared with inhibiting mTORC2 with Rictor siRNA (Figure 6A). The specificity and efficacy of the Raptor and Rictor siRNA knockdowns used in the experiments were ensured by measuring the steady-state levels of the Raptor (Figure 6B) and Rictor proteins (Figure 6C) with Western blot analysis, and the results indicate a more than 75% inhibition of the endogenous levels of Raptor using 50 nM of Raptor siRNA in ECs (Figure 6B) and a more than 95% inhibition of Rictor protein levels (Figure 6C) using the same amount of Rictor siRNA in ECs.

**Figure 7:**
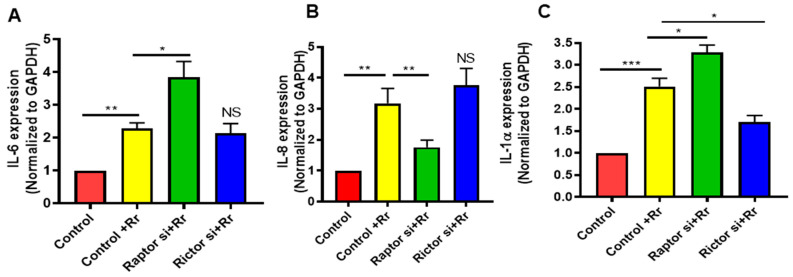
Effect of mTOR on cytokine expression in *R. rickettsii* (SS)-infected ECs. ECs were transfected with either control, Raptor, or Rictor siRNA 48 h prior to infection with *R. rickettsii* (SS) for 24 h. Total RNA was then isolated and cytokine expression (IL-6 (**A**), IL-8 (**B**), IL-1α (**C**)) was measured via qRT-PCR using a specific primer pair for each cytokine. Mock-infected cells are displayed as controls. Statistically significant changes are shown as * = *p* ≤ 0.05, ** = *p* ≤ 0.01, and *** = *p* ≤ 0.001; NS = not significant.

mTOR functions as a regulator of the innate immune responses of phagocytes (monocytes, macrophages, and myeloid dendritic cells) to pathogens by modulating cytokine and chemokine responses. We next determined whether mTOR (C1 and/or C2) inhibition further potentiates the pro-inflammatory phenotype of infected endothelial cells. Cells were transfected with Raptor or Rictor siRNA along with control siRNA 48 h prior to infection with *R. rickettsii* (SS); RNA was then isolated, and expression levels of IL-6, IL-8, and IL-1α were determined using their respective primer pairs. As expected, rickettsial infection significantly increased the levels of all three cytokines; the inhibition of mTORC1 by Raptor siRNA further increased the IL-6 (A) and IL-1α (C) mRNA but significantly reduced the IL-8 mRNA (B), while mTORC2 inhibition with Rictor siRNA had no effect on IL-6 and IL-8 mRNA but significantly reduced the IL-1α mRNA levels (Figure 7). These data suggest that the two different mTOR complexes, mTORC1 and mTORC2, regulate endothelial inflammation by differentially controlling the expression of proinflammatory cytokines.

## 4. Discussion

Endothelial cells constitute the inner lining of blood vessels and maintain organ homeostasis and communication with circulating blood. The vascular endothelium is preferentially targeted by many infectious agents, including rickettsia species. mTOR is an important regulator of autophagy and innate immune responses [20,25]. The present study demonstrates the differences in the activation of mTOR signaling by the avirulent (Iowa) and highly virulent (SS) strains of *R. rickettsii* during the infection of cultured human ECs mediated by both C1 (Raptor-mediated) and C2 (Rictor-based) mTOR complexes. In addition, mTOR inhibition promotes pathogen replication and regulates the expression of pro-inflammatory cytokines. It has been reported that *Listeria monocytogenes* [26], *Salmonella Typhimurium* [27], *Mycobacterium tuberculosis* [28], *Francisella tularensis* [29], and *Shigella flexneri* [30] utilize the mTOR pathway to enter, establish, and multiply in host cells. Pathogens utilize the host mTOR pathway and subvert metabolic processes to their advantage for their establishment and replication. mTOR, a member of the PI3-kinase family, may play a similar role in rickettsial pathogenesis, as PI3-kinase has been shown to promote the adhesion and invasion of *R. conorii* into host cells [31]. Our data demonstrate the differences in mTOR activation caused by avirulent vs. highly virulent bacterial strains, suggesting that the virulence of the bacteria may be involved in mTOR activation. Virulence factors have been shown to modulate mTOR stability in Leishmania-infected macrophages [32]. Thus, on the one hand, pathogens utilize mTOR to survive inside the host; on the other hand, host cells target mTOR signaling to facilitate pathogen clearance. Together, these findings suggest that intracellular bacteria have advanced in order to utilize essential metabolic machinery, the mTOR, for their own survival and replication, whereas the inhibition of mTOR triggers an antimicrobial immune response in favor of the host.

*R. rickettsii*, a member of the genus *Rickettsia*, exhibits a range of virulence, from acting as a harmless endosymbiont of arthropods to acting as an etiologic agent of severe disease. Despite the growing number of available genomes, little is known regarding the virulence determinants of rickettsiae. A multi-locus sequence alignment confirmed that avirulent *R. rickettsii* (Iowa) and highly virulent *R. rickettsii* (SS) exhibit a high degree of identity between their two genomes, but several strain-specific insertions, deletions, and SNPs were also identified, which may account for the difference in virulence. A major surface antigen, rickettsial outer membrane protein A (rOmpA), is severely truncated and exhibits a defect in processing another major surface antigen/autotransporter, rOmpB, in the Iowa strain [33]. The rickettsia ankyrin repeat protein 2 (RARP-2) protein in the avirulent Iowa strain contains a large internal deletion relative to the virulent Sheila Smith strain [34]. No differences have been observed in the ability to form actin tails on days 2 and 3 post-infection [24]. The replication pattern of both strains is identical, and the plaque sizes are indistinguishable, indicating that the two strains have similar rates of cell-to-cell spread [24]. These strain-specific truncation/deletions may contribute to the changes in the virulence of the Iowa and SS strains of *R. rickettsii*.

Many published reports demonstrate a clear link between mTOR and autophagy, an evolutionally conserved cellular degradation process in which cytoplasmic components such as organelles and invading microorganisms are sequestered and transferred to lysosomes for degradation and recycling [35]. Several intracellular pathogens have been shown to induce the autophagic process in host cells, which serves as an important host defense strategy for the clearance of microbes. On the other hand, pathogens hijack the host’s autophagic system to facilitate their survival and multiplication. Thus, the regulation of autophagy serves as a critical interface between pathogens and hosts. As intracellular bacteria, rickettsiae utilize host cell energy and take advantage of host metabolic resources. Our data indicate that the lipidation of LC3II is a marker of the accumulation of autophagosomes in SS-strain-infected ECs compared with the Iowa strain suggest the initiation of autophagic process by both strains, but rickettsiae establishing themselves and their intercellular spread clearly indicate their ability to evade autophagy to promote infection. Although autophagy plays a crucial role in host defense, intracellular microbes subvert and/or modify the autophagic process to facilitate their survival and replication [36,37]. It has been reported that *Rickettsia australis* initiates the autophagic process, as shown by its accumulation and co-localization with LC3 (+) autophagosomes in macrophages, but it evades the process, as suggested by the lack of change in levels of SQSTM1/p62 [38]. In another report, *Rickettsia parkeri* was shown to employ outer membrane protein B (rOmpB) to block the ubiquitylation of bacterial surface proteins, including OmpA, and subsequent recognition by autophagy receptors [39]. rOmpB is also required for the formation of the capsule-like *R. parkeri* to block autophagy in macrophages and to colonize mice because of its ability to promote autophagy evasion. OmpB has been shown to act as a protective shield to obstruct autophagy recognition, thereby providing a distinctive bacterial mechanism to evade antimicrobial autophagy [39]. Since rOmpA and rOmpB are truncated and display several defects in processing in the Iowa strain, it is likely that significant differences in the activation of mTOR via the avirulent and highly virulent strains of *R. rickettsii* are due to virulence factors.

In response to infection with intracellular microbes, proinflammatory and anti-inflammatory immune mechanisms are cross-regulated for host protection and limit pathogen-induced damage. In addition, innate and adaptive host responses trigger the proliferation of effector cells; induce the uptake of extracellular nutrients like free fatty acids, glucose, glutamine, and growth factors; and sense nutritional cues from the microenvironment to switch between metabolically inactive and active stages. Intracellular pathogens utilize host metabolic machinery for their survival and replication. There are reports that suggest that the systematic delivery of metabolism-altering agents such as rapamycin often has opposing actions: they limit pathogen replication but also alter the immune response to limit pathogen clearance. In the case of rickettsial infection, our data suggest that mTOR is activated and the autophagic response is initiated but not completed. Inhibiting mTOR with rapamycin reduces autophagy, which, in turn, favors bacterial replication like the data shown during mycobacterium infection [28].

As a sensor of cellular homeostasis, mTOR activity is often perturbed during infection, as intracellular pathogens induce amino acid starvation to trigger autophagy. Our findings show that rickettsiae likely exploit mTOR during infection to interfere with the autophagic response and promote replication in host ECs, as our data suggest that the inhibition of mTOR with low doses of rapamycin and Torin2 promotes rickettsial replication (Figure 5). Our data agree with a previously published report demonstrating that the pharmacological inhibition of mTOR signaling promotes the survival of *R. australis* in macrophages [38]. In contrast, the mTORC1-mediated regulation of autophagic responses in mouse macrophages offers a host defense mechanism against invasive bacteria like *M. tuberculosis*, *S. Typhimurium*, and *Pseudomonas aeruginosa*, promoting pathogen clearance and limiting microbial growth and replication [40,41,42,43]. *S. typhimurium* suppresses autophagy in mouse macrophages, enhancing intracellular pathogen survival [44]. In addition, HIV-1 downregulates mTOR-mediated autophagy and impairs the host’s innate and adaptive immune responses [45]. The inhibition of mTORC1 and mTORC2 with Raptor and Rictor siRNA promotes rickettsial replication, suggesting that the involvement of both complexes in the inhibition of autophagy in ECs during *Rickettsia* infection aligns with that of other pathogens. Intracellular pathogens like *Rickettsia* species multiply within the host cytosol and may utilize autophagy to increase cytosolic nutrients that support their survival and replication, as shown for *F. tularensis* [46]. It is likely that intracellular pathogens manipulate autophagic machinery to prevent xenophagic destruction while simultaneously utilizing autophagy-induced nutrients for their growth.

mTOR, as a versatile regulator of cellular functions, also acts as an important contributor to controlling inflammation by targeting NF-κB and the STAT family of transcription factors. Our previously published work clearly demonstrated that rickettsia infections of ECs activate NF-κB and STAT-1/3 and increase the secretion of cytokines and chemokines [1,2,3,47,48]. In response to pathogenic microbes, the increased production of various cytokines is a normal host immune response meant to recruit immune cells to the infection site for pathogen clearance. However, some pathogens, like *Staphylococcus aureus* and *Listeria monocytogenes*, utilize mTOR to promote the synthesis and secretion of anti-inflammatory cytokine IL-10 for their own benefit [49,50]. Our data demonstrate that mTOR inhibition increases the production of pro-inflammatory cytokines in rickettsia-infected cells, suggesting that mTOR may also contribute to the balance of pro-versus anti-inflammatory mediators during infection.

The innate immune system is critical for the maintenance of tissue homeostasis and responding to local/systemic perturbations induced by pathogenic insults. This rapid response must be metabolically supported to enable cell migration and proliferation and efficient production. Rickettsiae, based on their virulence factors, may utilize mTOR to manipulate the host metabolic machinery and mediate immunological consequences in favor of their needs, like survival and replication. In contrast, there are very few effective approaches that can regulate the host’s metabolism toward clearing microbes. One possibility may be that targeting metabolism with rapamycin or similar drugs often has contradictory actions, restricting pathogen replication but also limiting pathogen clearance by modulating the host’s immune responses.

## Data Availability

Data are contained within the article and supplementary materials.

## Supplementary Materials

The following supporting information can be downloaded at https://www.mdpi.com/article/10.3390/microorganisms12020296/s1: Figure S1: Growth of Iowa and Sheila Smith (SS) strains of *R. rickettsii* in endothelial cells. Table S1: List of primers used in this study.
