# Supplementary material for: Endothelial Mechanistic Target of Rapamycin Activation with Different Strains of R. rickettsii: Possible Role in Rickettsial Pathogenesis
Source: Microorganisms. 2024 Jan 30;12(2):296. doi: 10.3390/microorganisms12020296 (PMC10892065; doi:10.3390/microorganisms12020296)
Supplement: Supplementary file 1 [file microorganisms-12-00296-s001.zip › microorganisms-2814547-supplementary.pdf]

Supplementary Data:

Figure 1. Growth of Iowa and Sheila Smith (SS) strain of *R. rickettsii* in endothelial cells

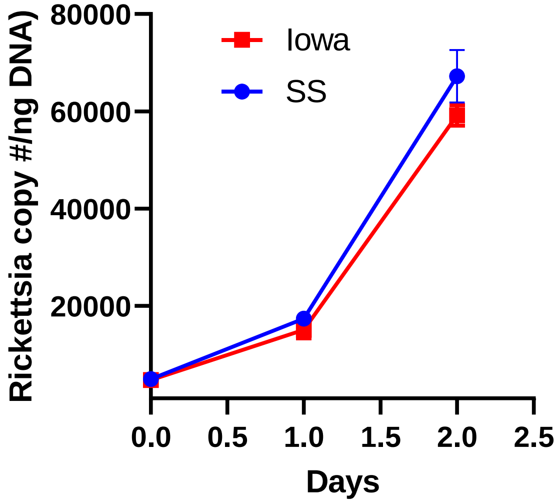

Confluent endothelial cells were infected with *R. rickettsii* (SS and Iowa) with similar number of bacteria, after 24 and 48h, DNA was isolated and rickettsiae copy number was measured using gltA primer pair.

Supplemental Table:

Supplementary Table: List of primers used in this study

**Gene**	**Orientation**	**Primer sequence (5'-3')**
gltA	Forward	TCACTGCTTCATTAGCCCCG
	Reverse	AGTTCGGATTGCTGGCTCAT
ompA	Forward	CCTGCCGATAATTATACAGGTTTA
	Reverse	GTTCCGTTAATGGCAGCAT
18S	Forward	GTAACCCGTTGAACCCCATT
	Reverse	CGCTACTACCGATTGGATGG
GAPDH	Forward	CTGGTAAAGTGGATATTGTTGCCAT
	Reverse	TGGAATCATATTGGAACATGTAAACC
IL-6	Forward	CGCTGACGGAGTACAAGTG
	Reverse	GAAGGCAGCAGGCAACAC
IL-8	Forward	AGACAGCAGAGCACACAAGC
	Reverse	AGGAAGGCTGCCAAGAGAG
IL-1α	Forward	TGGTAGTAGCAACCAACGGGA
	Reverse	ACTTTGATTGAGGGCGTCATTC
